# Precision Monitoring Strategies for Chemotherapy-Induced Cardiotoxicity: A Review of Molecular Indicators for Early Detection and Risk Stratification

**DOI:** 10.31083/RCM45852

**Published:** 2026-02-25

**Authors:** Ying Kong, Ruihong He, Haiqing He, Lijuan Liao, Chao Wu, Xuanying Chen, Xiaoping Peng

**Affiliations:** ^1^Department of Pharmacy, The First Affiliated Hospital, Jiangxi Medical College, Nanchang University, 330006 Nanchang, Jiangxi, China; ^2^Department of Cardiology, The First Affiliated Hospital, Jiangxi Medical College, Nanchang University, 330006 Nanchang, Jiangxi, China; ^3^Department of Pharmacy, The First People's Hospital of NanKang District, 341400 Ganzhou, Jiangxi, China; ^4^Department of Pharmacy, The First People's Hospital of Longnan City, 341700 Longnan, Jiangxi, China; ^5^Department of Pharmacy, Ganjiang New Area People’s Hospital, 330114 Nanchang, Jiangxi, China

**Keywords:** cardiotoxicity, chemotherapy, biomarkers, risk stratification, surveillance, heart failure

## Abstract

Chemotherapy-induced cardiotoxicity (CIC) is an increasingly recognized complication in cancer survivors, particularly with anthracyclines, human epidermal growth factor receptor 2 (HER2) inhibitors, vascular endothelial growth factor (VEGF) inhibitors, and immune checkpoint inhibitors. CIC may present acutely, chronically, or as a delayed condition, with phenotypes ranging from asymptomatic myocardial dysfunction to heart failure, arrhythmias, and myocarditis. This narrative review aimed to summarize the latest evidence on the pathogenesis of CIC and evaluate traditional and emerging biomarkers for early detection and risk stratification. We comprehensively reviewed the literature related to the pathogenesis and biomarkers of CIC, focusing on studies that examined oxidative stress, DNA damage, mitochondrial dysfunction, inflammation, and immune activation. The five most frequently reported mechanisms in CIC toxicity were oxidative stress, DNA damage, mitochondrial dysfunction, inflammation, and immune activation. Traditional biomarkers, such as cardiac troponin and natriuretic peptides, have been shown to aid in early detection; however, these biomarkers are limited by specificity and timing. Emerging biomarkers, including inflammatory cytokines, fibrosis-related proteins, extracellular vesicles, and non-coding RNAs, demonstrate greater sensitivity and potential for earlier risk stratification. However, study heterogeneity and limited validation across populations hinder clinical translation. Thus, integrating biomarkers with imaging modalities and standardized protocols may enhance personalized surveillance of CIC toxicity. Large prospective studies and standardized frameworks are essential. Hence, a multiparametric approach combining molecular, functional, and computational tools may define future precision monitoring for CIC toxicity.

## 1. Introduction

The global survival rate of cancer patients has markedly improved in recent years, with the continuous optimization of tumor screening and significant advances in cancer therapies. However, this progress has been accompanied by a steady increase in cardiovascular complications associated with anticancer treatments, particularly chemotherapy-induced cardiotoxicity (CIC) [1, 2, 3, 4]. CIC encompasses various cardiac manifestations, including arrhythmias, ischemic cardiomyopathy, and chronic heart failure, and has become a leading cause of cardiovascular morbidity and mortality among long-term cancer survivors [3, 5, 6]. Anthracycline-based drugs and human epidermal growth factor receptor 2 (HER2)-targeted therapies are the most common chemotherapeutic agents associated with cardiac toxicity and have been extensively investigated [7, 8, 9].

Early detection of CIC is crucial for determining timely treatment modifications and improving both cardiovascular and oncological outcomes [10, 11]. Myocardial injury associated with chemotherapy often precedes overt structural or functional abnormalities detectable by imaging, and delayed diagnosis may lead to irreversible cardiac dysfunction [12, 13, 14]. Therefore, the development of diagnostic tools that are sensitive, specific, and capable of dynamic monitoring is crucial for improving prognosis and quality of life [11, 15, 16].

Compared with traditional imaging modalities such as echocardiography and multigated acquisition (MUGA) scans, circulating biomarkers offer practical advantages, including the ease of use, reproducibility, and the ability to detect subclinical myocardial injury at the cellular level [10, 14, 17]. Troponins and brain natriuretic peptides (BNPs), the most widely used markers for cardiotoxicity, are recommended by some guidelines for cardiac monitoring during cancer therapy [11, 15]. Ongoing research on emerging biomarkers, including inflammatory cytokines, fibrosis-associated molecules, circulating microRNAs, and extracellular vesicles (EVs), has yielded promising tools for precise stratification and early intervention in CIC [18, 19, 20, 21, 22, 23, 24].

This review focuses on the clinical utility and mechanistic relevance of circulating biomarkers to detect CIC. We summarize their functional classifications, discuss their involvement in the molecular pathogenesis of CIC, and evaluate their potential for clinical translation. This review aims to support the development of an effective and standardized monitoring system for cancer treatment-related cardiac toxicity.

## 2. Chemotherapy-Induced Cardiotoxicity (CIC)

### 2.1 Definition and Clinical Classification of CIC

According to the latest guidelines from the European Society of Cardiology (ESC) and the International Society of Cardiac Oncology (IC-OS), CIC exhibits a heterogeneous clinical spectrum and is classified by its timing of onset: acute, chronic, or late-onset forms [1, 3, 4]. This temporal classification, though clinically useful, does not fully capture the expanding phenotypes of cardiotoxicity arising from diverse oncologic regimens [5, 6, 25].

#### 2.1.1 Acute CIC

Acute CIC occurs during chemotherapy or within a few days of initiation of treatment. Although rare (<1%), it is mainly characterized by transient arrhythmias, acute myocarditis, or short-term declines in myocardial contractility [8, 23]. It is commonly associated with anthracyclines, pathological changes which include myocardial edema, mitochondrial damage, and free radical-mediated oxidative stress [19, 26].

#### 2.1.2 Chronic CIC

Chronic CIC, often subdivided into early and late-onset forms, typically manifests within months after chemotherapy. Early-onset chronic CIC has an incidence of 1.6%–2.1% [5, 6] and may eventually progress to left ventricular (LV) systolic dysfunction or dilated cardiomyopathy [9]. Traditionally considered dose-dependent and irreversible, recent studies suggest that subclinical dysfunction can occur at lower anthracycline doses than previously recognized [16, 27].

#### 2.1.3 Late-Onset Chronic CIC

Late-onset CIC emerges years to decades post-treatment and results in significant concerns regarding long-term survival [3, 4]. It includes progressive heart failure, ischemic heart disease, arrhythmias, and valvular abnormalities [2]. Despite its clinical significance, long-term surveillance protocols remain inadequately implemented [6, 25].

To standardize risk stratification, IC-OS and ESC guidelines recommend grading CIC by severity using parameters such as LV ejection fraction (LVEF), global longitudinal strain (GLS), and elevated cardiac biomarkers. CIC is classified as mild (LVEF ≥50% with GLS decrease >15%, and elevated troponins), moderate (LVEF 40%–49%), or severe (LVEF <40%) [11, 17, 19].

CIC involves changes beyond systolic dysfunction including arrhythmias, myocarditis, and microvascular abnormalities. Therefore, reliance on LVEF alone is insufficient for comprehensive surveillance [28, 29].

### 2.2 Cardiotoxic Chemotherapeutic Agents and Mechanisms

**Fig. 1.**
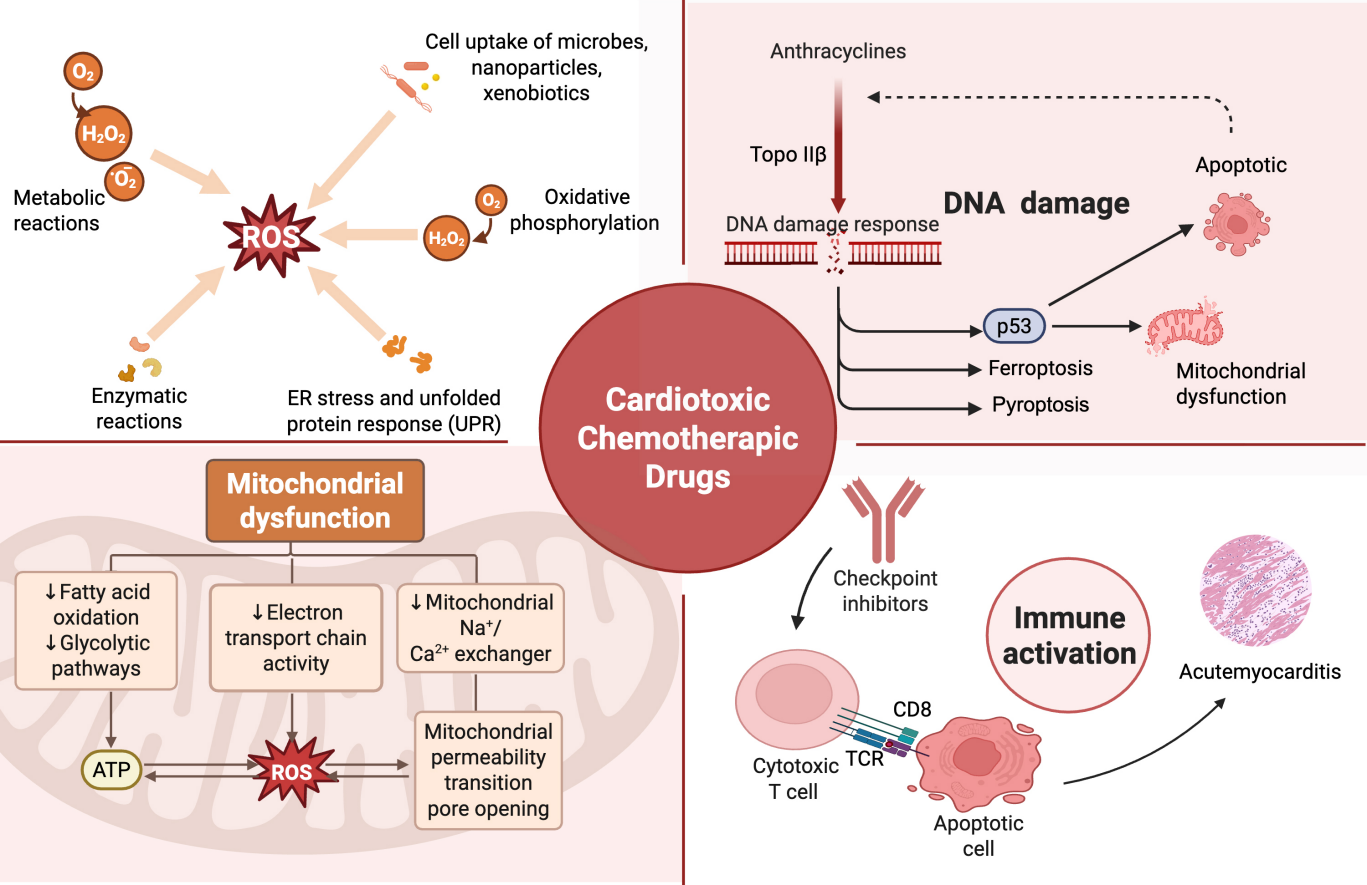
**Mechanistic pathways of cardiotoxic chemotherapeutic agents**. The major molecular mechanisms by which chemotherapeutic drugs induce cardiotoxicity. (1) Reactive oxygen species overproduction, triggered by metabolic reactions and oxidative phosphorylation, leads to endoplasmic reticulum stress and cellular injury; (2) mitochondrial dysfunction, resulting from impaired fatty acid oxidation, electron transport chain activity, and abnormal mitochondrial permeability transition pore opening; (3) DNA damage, particularly associated with anthracyclines via Topoisomerase IIβ inhibition, activates p53-dependent apoptotic pathways; and (4) immune activation, as seen with immune checkpoint inhibitors, promotes cytotoxic T cell–mediated myocarditis and apoptosis, ferroptosis through lipid peroxidation, and pyroptosis mediated by inflammasome activation. These mechanisms collectively contribute to the structural and functional cardiac impairments observed in chemotherapy-induced cardiotoxicity (CIC). ROS, reactive oxygen species; TCR, T-cell receptor. Fig. 1 was created with BioRender.

The pathogenesis of CIC is multifactorial and varies by drug class. Nonetheless, several common mechanisms, including oxidative stress, DNA damage, mitochondrial dysfunction, inflammation, and immune activation, are common across the various agents [10, 14, 30, 31]. The mechanistic pathways of cardiotoxic chemotherapeutic agents are summarized in Fig. 1.

#### 2.2.1 Oxidative Stress

Oxidative stress is a hallmark mechanism in the pathogenesis of CIC, particularly for anthracyclines such as doxorubicin (DOX) [32]. These agents undergo redox cycling within cardiomyocytes, producing excessive reactive oxygen species (ROS) that overwhelm cardiac antioxidant defenses [1, 12, 19]. The myocardium is particularly susceptible due to low levels of catalase and superoxide dismutase [1, 12]. ROS-induced lipid, protein, and DNA damage triggers cardiomyocyte apoptosis and necrosis [1, 12]. Recent transcriptomic and proteomic analyses confirm that antioxidant gene expression is significantly downregulated following DOX exposure. Although the mechanisms are well-established, antioxidant-based interventions have shown inconsistent clinical efficacy, highlighting that oxidative stress is an upstream contributor rather than the sole determinant of injury [7]. Other agents, including tyrosine kinase inhibitors and vascular endothelial growth factor (VEGF) inhibitors, also promote ROS generation indirectly through mitochondrial and endothelial dysfunction, especially under hypertensive conditions [10, 11, 26].

#### 2.2.2 DNA Damage

DNA damage is another key mechanism, particularly for drugs that target topoisomerases or form DNA adducts. DOX inhibits topoisomerase IIβ in cardiomyocytes, inducing double-stranded DNA breaks and activating DNA damage response (DDR) pathways such as ataxia telangiectasia mutated (ATM) and p53 [5, 26]. In addition to the induction of apoptosis, accumulating evidence indicates that DNA lesions can also trigger non-apoptotic regulated forms of cell death. Ferroptosis, characterized by iron-dependent lipid peroxidation, has been implicated in anthracycline-induced cardiomyopathy, and pyroptosis, mediated by inflammasome activation and caspase-1 signaling, is linked to DNA injury resulting in the release of inflammatory cytokines [22, 33]. Necroptosis has also been reported under conditions of sustained DDR and mitochondrial failure. These overlapping pathways demonstrate that chemotherapy-induced DNA damage leads to a spectrum of cardiomyocyte death modalities, which contribute to cardiac dysfunction. Importantly, the role of topoisomerase IIβ distinguishes cardiotoxicity from tumoricidal effects, which depend on topoisomerase IIα, thus offering a theoretical window for selective protection [5, 27]. However, few clinical strategies currently exist to mitigate DNA damage in the heart without compromising antitumor efficacy. Dexrazoxane, an iron chelator, mitigates this damage by stabilizing topoisomerases and limiting ROS generation, but its clinical use remains limited due to concerns over interference with antitumor efficacy [5, 16, 27].

#### 2.2.3 Mitochondrial Dysfunction

Mitochondrial dysfunction occurs when chemotherapy impairs oxidative phosphorylation, disrupts membrane potentials, and induces mitochondrial DNA (mtDNA) damage [12, 17, 20, 23]. Single-cell RNA sequencing and mitochondrial stress assays have demonstrated a rapid decline in mitochondrial energy metabolism following exposure to DOX [11, 17, 19, 34]. Mitochondria are also involved in multiple injury pathways involving ROS, DNA damage, and apoptosis, making them important targets in the pathophysiology of CIC [24, 35, 36]. Although mitochondrial-targeted antioxidants are in preclinical development, none have yet been clinically validated [11, 17, 23]. Mitochondrial dysfunction manifests as impaired oxidative phosphorylation, leading to a critical deficit in ATP production that compromises cardiomyocyte contractility and calcium handling, thereby directly contributing to left ventricular dysfunction. Injured mitochondria release excessive ROS and pro-apoptotic factors, triggering programmed cell death and fibrotic remodeling. The release of mtDNA results in a damage-associated molecular pattern, which upon engagement with Toll-like receptor 9 (TLR9) on immune cells, instigates a pro-inflammatory cytokine response including interleukin (IL)-1β and IL-6 that predisposes to myocardial inflammation and remodeling. Collectively, these interconnected pathways including depletion of energy sources, oxidative stress, cell death, and sterile inflammation result in the development of specific cardiac pathologies such as dilated cardiomyopathy and heart failure, establishing mitochondrial integrity as a central mediator in the pathogenesis of CIC [37].

#### 2.2.4 Immune Activation

Immune activation is particularly relevant to immune checkpoint inhibitors (ICIs) such as anti-PD-1, anti-CTLA-4, and results in autoimmune myocarditis characterized by lymphocytic infiltration, myocyte necrosis, and elevated cardiac biomarkers. Although rare (<1%), ICI-associated myocarditis has a reported mortality of >40% and may occur early after the initiation of therapy [38]. ICIs enhance T-cell responses by blocking co-inhibitory signals, potentially disrupting tolerance to cardiac self-antigens. Murine models and human biopsy samples show CD8+ T-cell predominance and shared T-cell receptor (TCR) clonotypes between cardiac and tumor tissue, supporting an autoimmune etiology [38]. However, the unpredictable nature of immune activation remains a clinical challenge. No reliable predictive biomarkers exist, and routine surveillance remains controversial.

#### 2.2.5 Inflammatory Activation

Inflammatory activation of nuclear factor kappa-light-chain-enhancer of activated B cells (NF-κB), mitogen-activated protein kinase (MAPK), and NOD-like receptor thermal protein domain associated protein 3 (NLRP3) signaling pathways [19] promotes cytokine release, leukocyte infiltration, and extracellular matrix remodeling. Macrophage-mediated inflammation has been implicated in persistent cardiac fibrosis in anthracycline models. Single-cell transcriptomics reveal that even non-myocytes shift to proinflammatory phenotypes, contributing to multicellular injury [36, 39]. Although preclinical anti-inflammatory interventions are promising, concerns regarding immune suppression and tumor progression limit their clinical application.

Although these mechanisms provide insight into the pathogenesis of CIC, considerable overlap exists. Moreover, current preclinical models inadequately reflect the complexity introduced by host factors such as aging, metabolic disease, or genetic predisposition, each of which determines the individual susceptibility to cardiotoxicity.

#### 2.2.6 Crosstalk and Synergistic Interactions Among Various Mechanisms

**Fig. 2.**
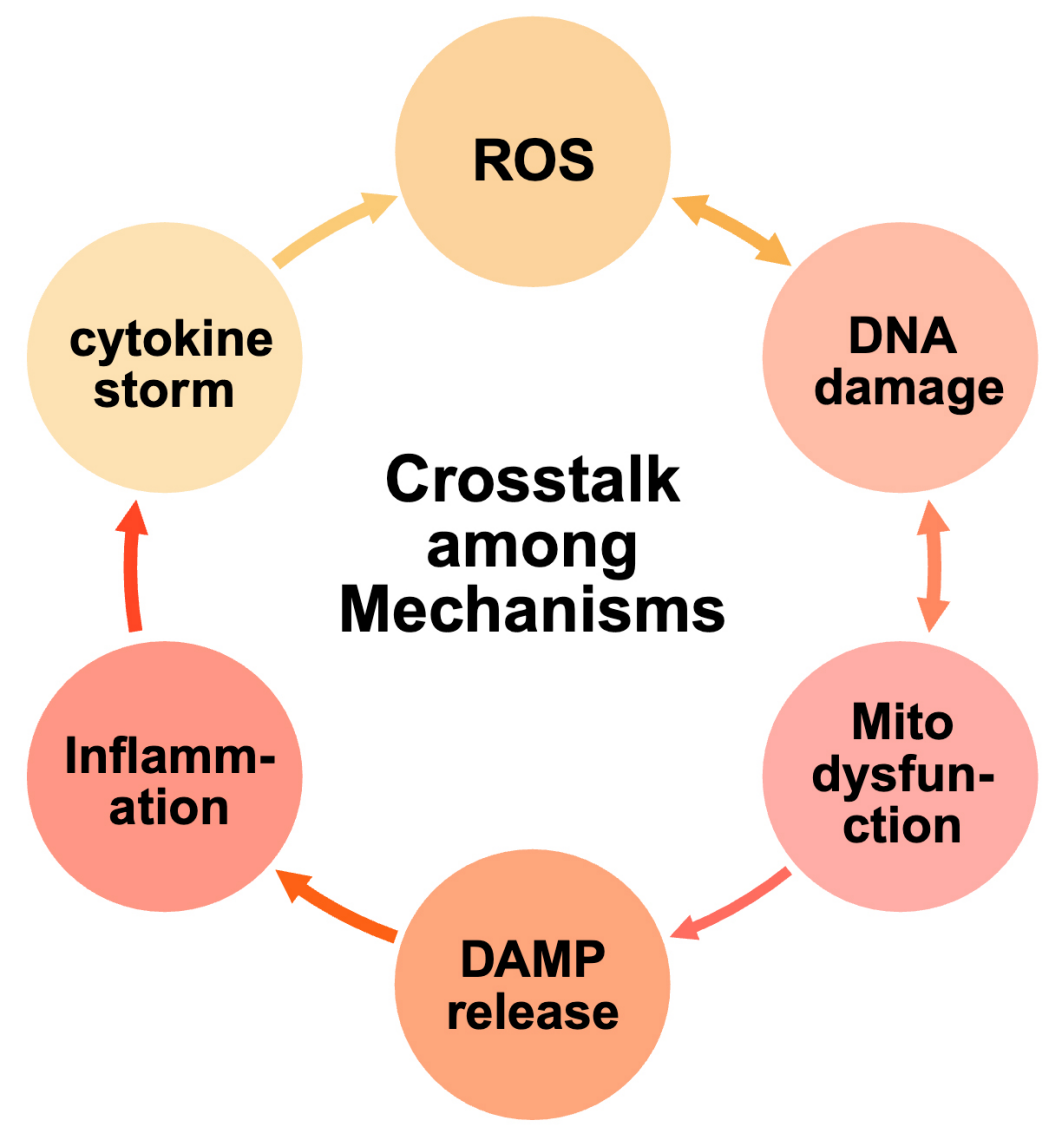
**Crosstalk among mechanisms of chemotherapy-induced cardiotoxicity**. Oxidative stress, DNA damage, mitochondrial dysfunction, inflammation, and immune activation form a tightly interconnected network driving cardiotoxicity. Excessive ROS promotes breaks in DNA strands and mitochondrial injury, while mitochondrial dysfunction increases ROS production and releases damage-associated molecular patterns (DAMPs), further activating inflammatory cascades. Inflammatory cytokines intensify oxidative stress and structural remodeling, whereas immune checkpoint inhibitor–induced T-cell activation reinforces inflammation and causes direct cardiomyocyte injury. These synergistic interactions establish a vicious cycle of apoptosis, necrosis, and fibrosis, ultimately leading to cardiac dysfunction.

The pathogenic mechanisms of chemotherapy-induced cardiotoxicity rarely occur in isolation, rather, they constitute a tightly interconnected network of mutually reinforcing processes, as shown in Fig. 2. For instance, oxidative stress not only damages cardiomyocyte DNA but also triggers mitochondrial dysfunction by impairing electron transport chain activity and the generation of ATP [22, 40]. Mitochondrial dysfunction exacerbates oxidative stress by releasing additional ROS and reactive nitrogen species, resulting in a vicious cycle of cellular injury [33, 41].

Simultaneously, DNA damage activates the p53 pathway, which promotes apoptosis, contributing to mitochondrial destabilization, thereby increasing ROS production and apoptotic signaling cascades [40]. Immune checkpoint inhibitor–induced T-cell activation illustrates how immune dysregulation contributes to this process. Activated cytotoxic lymphocytes release interferon-γ and perforin–granzyme complexes, resulting in direct cardiomyocyte damage and fueling the release of inflammatory cytokines [33, 42]. This immune amplification loop synergizes with mitochondrial and oxidative injury, creating a self-perpetuating cycle of cardiotoxicity. These interactions highlight that chemotherapy-induced cardiotoxicity emerges not from discrete insults but from the convergence of oxidative, genetic, metabolic, inflammatory, and immune pathways.

## 3. Traditional Biomarkers

### 3.1 Imaging Markers

Echocardiography, particularly measurement of LVEF, remains the standard method for monitoring CIC [24, 43]. However, LVEF reflects late-stage cardiac damage which may not decline until irreversible injury has occurred [44]. GLS, which quantifies myocardial deformation, offers earlier detection of subclinical dysfunction [14, 45]. A >15% relative reduction in GLS during chemotherapy predicts subsequent decline in cardiac function [24, 45], making it a more sensitive early marker of subclinical dysfunction. Nonetheless, GLS has limitations, including inter-operator variability and influence by a patient’s hydration status and comorbidities. In many clinical settings, frequent imaging is impractical [46, 47]. Furthermore, imaging cannot detect biochemical or molecular alterations that precede functional changes, underscoring the need for more sensitive and accessible biomarkers [43].

### 3.2 Cardiac Troponins

Cardiac troponins, particularly troponin I (cTnI) and troponin T (cTnT), are highly specific for myocardial injury since they are expressed only in cardiomyocytes [43]. In CIC, troponin elevation may occur within hours to days after exposure to agents such as anthracyclines or trastuzumab [7, 31], often preceding changes detectable by imaging. Persistent troponin elevation is associated with an increased risk of LV dysfunction and heart failure [9]. Serial measurements demonstrate that patients with continuous troponin elevation are more likely to develop reduced ejection fraction or symptomatic heart failure [6]. Troponins thus serve as early indicators of subclinical injury and tools for patient risk stratification. However, their use in oncology remains limited due to a lack of standardized measurement protocols. Studies vary in timing, frequency, and cut-off thresholds, which are often influenced by assay sensitivity, patient age, or baseline cardiac status [6]. These inconsistencies limit their routine use and can complicate interpretation, particularly in borderline cases.

### 3.3 Natriuretic Peptides

BNP and N-terminal pro-brain natriuretic peptide (NT-proBNP) are released in response to ventricular stretch and pressure overload. Unlike troponins, which reflect direct injury, natriuretic peptides indicate myocardial stress or dysfunction, especially in heart failure [11, 48]. Elevated levels have been observed in patients treated with anthracyclines, HER2 inhibitors, and tyrosine kinase inhibitors [49] and are more useful in detecting chronic or delayed cardiotoxicity. Persistent NT-proBNP elevation during chemotherapy correlates with poor long-term cardiac outcomes, even when LVEF remains normal [49, 50, 51]. However, their specificity is limited, as levels can rise due to renal impairment, infection, anemia, or advanced age. Thus, interpretation requires correlation with the patients’ current clinical condition [6].

Troponins, natriuretic peptides, and echocardiographic markers such as GLS are essential for CIC monitoring. However, their limitations underscore the need for novel biomarkers that are accurate, accessible, and predictive of early-stage injury across diverse populations.

## 4. Emerging Biomarkers

Recent advances in understanding CIC pathophysiology have led to the identification of emerging biomarkers that reflect early molecular changes. These markers may offer more precise risk stratification and individualized monitoring.

### 4.1 Inflammatory and Oxidative Stress Markers

**Fig. 3.**
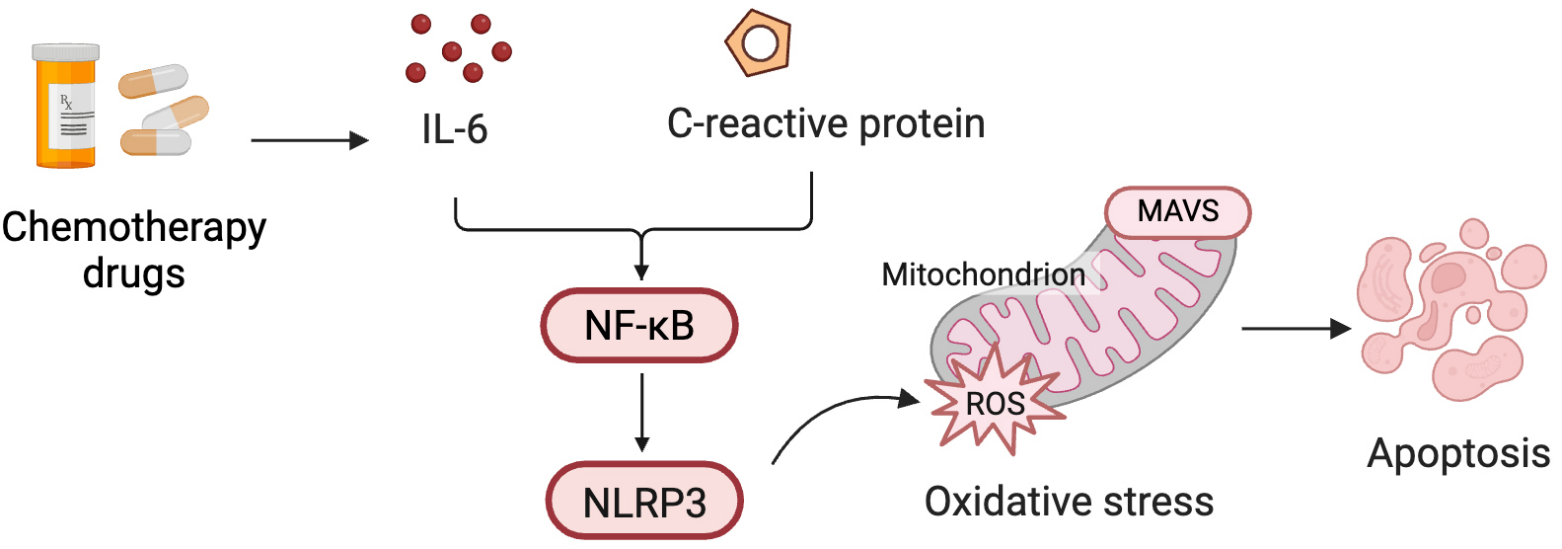
**Inflammatory pathways mediated by CIC injury**. Chemotherapy drugs induce the release of inflammatory mediators such as interleukin (IL)-6 and C-reactive protein (CRP), which activate the nuclear factor kappa-light-chain-enhancer of activated B cells (NF-κB) and NOD-like receptor pyrin domain-containing 3 (NLRP3) signaling pathways. This activation promotes mitochondrial oxidative stress via accumulation of reactive oxygen species (ROS), leading to cardiomyocyte apoptosis and structural cardiac remodeling. MAVS (mitochondrial antiviral signaling protein) is involved in mitochondrial dysfunction under conditions of oxidative stress. Fig. 3 was created with BioRender.

Inflammation plays a critical role in the initiation and amplification of myocardial injury in CIC [9, 19]. Early activation of inflammatory pathways can sensitize the myocardium to oxidative stress and mitochondrial dysfunction, which facilitates irreversible remodeling. Several biomarkers have been proposed to reflect this inflammatory–oxidative axis. Myeloperoxidase (MPO), secreted by neutrophils, has been associated with early cardiac injury during anthracycline therapy [13, 23]. Elevated levels of C-reactive protein (CRP) and IL-6 are linked to a decline in LVEF and adverse cardiovascular outcomes, particularly in patients with comorbidities [52, 53, 54]. Growth differentiation factor-15 (GDF-15), a mitochondrial stress-responsive cytokine, has emerged as a promising marker of cardiac strain and systemic toxicity [55]. However, most supporting studies are observational and use variable assay methods, limiting comparability [9, 13]. The causal role of these markers and their utility in routine practice remains to be established. Inflammatory pathways mediated by chemotherapy drugs are shown in Fig. 3.

### 4.2 Fibrosis and Remodeling-Related Markers

Cardiac fibrosis and extracellular matrix remodeling are central to chronic and late-onset CIC, offering biomarkers that provide insights into disease progression beyond acute injury [11]. Galectin-3, a β-galactoside-binding lectin involved in fibroblast activation and collagen synthesis, has been linked to long-term adverse cardiac outcomes in both heart failure and patients undergoing chemotherapy [22]. Soluble ST2 (sST2), a member of the IL-1 receptor family, is upregulated in response to myocardial stretch and inflammation, with elevated levels correlating with myocardial fibrosis and increased mortality in heart failure patients [11]. In patients receiving chemotherapy, early sST2 elevation has been observed prior to a decline in LVEF, demonstrating its potential as a predictive biomarker of impending myocardial dysfunction [18, 56].

In models of heart failure with preserved ejection fraction (HFpEF), both galectin-3 and sST2 are particularly relevant, as these patients typically have no obvious reduction in LVEF, however they ultimately develop progressive diastolic dysfunction and myocardial stiffening [56, 57, 58]. Their expression reflects the burden of fibrosis rather than contractile failure, highlighting their utility in detecting non-systolic CIC phenotypes.

Despite these promising findings, the clinical use of fibrosis markers faces challenges. Their levels are influenced by systemic conditions such as renal dysfunction and malignancy [12], and their relatively long half-lives and broad expression limit temporal specificity. Nonetheless, when interpreted along with troponin levels and imaging modalities such as GLS, galectin-3 and sST2 may enhance diagnostic accuracy.

### 4.3 Novel Molecular Biomarkers

#### 4.3.1 Extracellular Vesicles (EVs)

EVs, including exosomes and microvesicles, are lipid-bound carriers of proteins, microRNAs, and other molecules [11, 25, 59]. Cardiomyocyte-derived EVs in CIC have shown potential for detecting early stress, mitochondrial injury, and apoptosis [60]. Their stability in the circulation and specificity offer promise for use as “liquid biopsy” tools [24, 61]. However, technical issues in isolation, quantification, and standardization remain major barriers to their clinical application [25]. Most studies remain in early-stage discovery; thus, EVs are not yet included in guideline-recommended monitoring strategies [25, 47].

#### 4.3.2 Circulating MicroRNAs and LncRNAs

MicroRNAs (miR-1, miR-21, miR-133a, and miR-208a) and long non-coding RNAs (lncRNAs) regulate cardiac gene expression and have been associated with early cardiotoxic changes during anthracycline therapy [36, 39]. These alterations often precede biomarker or imaging abnormalities. Similarly, dysregulation of certain lncRNAs has been observed in both animal models and patient samples, suggesting their involvement in apoptosis, oxidative stress, and fibrosis. However, their use is limited by assay variability, individual genetics, and tumor heterogeneity, and thus they are not yet standardized for clinical use [36, 39].

#### 4.3.3 Emerging Protein Biomarkers

GDF-15, a stress-responsive cytokine, is increasingly recognized as a marker of mitochondrial dysfunction, inflammation, and cachexia in CIC [18, 55]. Elevated GDF-15 levels have been reported in patients receiving DOX or immune checkpoint inhibitors and are associated with systemic toxicity and cardiac stress. Similarly, placental growth factor (PlGF), a member of the VEGF family, reflects endothelial injury and vascular dysfunction [22]. Preliminary studies suggest a correlation between PIGF levels and cardiotoxicity, particularly in patients receiving anti-angiogenic therapies.

These proteins involve pathophysiological pathways beyond those detected by conventional biomarkers, offering mechanistic insight and potential for early detection of multisystem toxicity. However, most findings remain exploratory. Large-scale, prospective studies are needed to validate their clinical relevance and establish their role in routine cardio-oncology practice.

The emerging and traditional biomarkers for CIC are compared in Table 1.

**Table 1.** **Comparison of emerging and traditional biomarkers chemotherapy-induced cardiotoxicity (CIC)**.

Category	Source	Clinical relevance	Advantages	Limitations
Cardiac troponins (cTnI/T)	Released from injured cardiomyocytes	Early detection of cardiomyocyte necrosis and prediction of CIC risk	High specificity, rapid response time	Lack of standardized cut-offs; transient elevations may be misleading
Brain natriuretic peptide (BNP)/NT-proBNP	Secreted in response to ventricular wall stress	Reflects volume overload and chronic ventricular pressure; used for monitoring delayed toxicity	Suitable for serial monitoring; correlates with heart failure risk	Low specificity; affected by age, renal function, and anemia
Global longitudinal strain (GLS)	Echocardiographic assessment of myocardial deformation	Detects subclinical left ventricular (LV) dysfunction earlier than LV ejection fraction (LVEF)	Non-invasive; high sensitivity	Operator- and image-dependent; limited accessibility for frequent use
Inflammatory markers (CRP, IL-6, myeloperoxidase (MPO))	Released during immune activation and oxidative stress	Serve as early indicators of myocardial inflammation and injury	Capture subclinical inflammatory activity	Low specificity; influenced by systemic conditions
Fibrosis markers (Galectin-3, soluble ST2 (sST2))	Secreted by fibroblasts and inflammatory cells	Associated with myocardial fibrosis and late-stage CIC	Linked to heart failure with preserved ejection fraction (HFpEF) and long-term prognosis	Widely expressed; limited cardiac and temporal specificity
Extracellular vesicles (EVs)	Released by cardiomyocytes and other cells	Transport molecular signatures of early cardiac injury; potential for liquid biopsy	High stability; may enable real-time monitoring	Isolation and quantification are technically challenging; lack of standardization
Circulating ncRNAs (miRNA, lncRNA)	Secreted into circulation under stress	Regulate gene expression related to cardiac injury; predict early CIC	Detectable before functional changes appear	Assay platform variability; high inter-individual heterogeneity
Emerging proteins (growth differentiation factor-15 (GDF-15), placental growth factor (PlGF))	Expressed under stress, inflammation, or endothelial injury	Reflect systemic toxicity and cardiac stress responses	Indicate multisystem involvement at early stages	Limited validation; currently in exploratory research phase

## 5. Clinical Application and Translational Considerations

As biomarker research progresses, translating findings into clinical practice remains a key challenge. Although several biomarkers show mechanistic promise, their practical utility depends on reproducibility, feasibility, and integration into oncology care pathways [45].

### 5.1 Multi-Biomarker Integration Strategies

No single biomarker adequately reflects the complex, multi-staged progression of CIC. Integrative approaches that combine biomarkers with imaging modalities have emerged to improve diagnostic precision. For example, dynamic troponin changes paired with GLS can detect subclinical injury before a decline in LVEF [45]. Similarly, elevation of inflammatory markers such as IL-6 and GDF-15 with mild GLS decline may indicate early, potentially reversible myocardial damage [3, 22].

In patients undergoing immunotherapy or multi-targeted regimens, relying solely on LVEF may underestimate the risk of injury. Multi-marker strategies may enhance sensitivity and better capture individual risk [22]. Nonetheless, a major limitation is the lack of standardized testing protocols. Suggested approaches include dynamic biomarker evaluation at baseline, after each chemotherapy cycle, and within 1–3 months post-treatment, particularly for markers with known variability such as troponin and NT-proBNP [7, 17, 43]. Standardized timepoints also facilitate development of risk scoring models such as the Troponin–GLS–NT-proBNP triad framework [17, 62, 63]. Future research must also define thresholds for intervention—for instance, whether a >15% reduction in GLS along with sustained troponin elevation should prompt a delay in treatment remains to be validated [64, 65].

### 5.2 Population-Specific Considerations

Biomarker interpretation must be personalized to account for population-specific risk [7, 17]. High-risk groups such as those with pre-existing cardiovascular disease, advanced age, or concurrent radiotherapy are more likely to experience early manifestations of CIC. In these patients, highly sensitive markers such as troponin and sST2 may be more predictive, and dynamic monitoring is preferred over isolated measurements [11].

In contrast, biomarker elevations in low-risk populations—particularly those influenced by non-cardiac factors such as IL-6 or BNP—require cautious interpretation and must be correlated with imaging and clinical data [11]. Pediatric and adolescent cancer patients, with developing cardiac structures that are still being developed, may also demonstrate distinct biomarker patterns. For example, high-sensitivity troponin has shown strong predictive value for early cardiomyocyte injury in children receiving anthracyclines, often rising before echocardiographic changes become evident. Moreover, circulating natriuretic peptides and emerging biomarkers such as miRNAs and GDF-15 appear to provide complementary insights into long-term cardiac vulnerability in pediatric survivors [11]. Elderly patients, in contrast, present additional challenges due to baseline myocardial remodeling, impaired renal clearance, and a higher prevalence of comorbidities [66]. Natriuretic peptides are frequently elevated in this group even before chemotherapy, reducing their specificity for cardiotoxicity. Nonetheless, high-sensitivity troponins retain incremental predictive value, and their serial measurement improves discrimination between pre-existing cardiac dysfunction and new-onset CIC [67]. In patients with comorbid conditions such as hypertension, diabetes, or chronic kidney disease, inflammatory and metabolic biomarkers may be persistently elevated, complicating their interpretation [67].

Tumor type and treatment regimens further influence biomarker profiles. For example, GDF-15 levels are significantly elevated in immune-checkpoint inhibitor-associated myocarditis, whereas troponin may lack sensitivity in this context [3, 18, 55].

In summary, successful clinical translation requires not only individual biomarker validation but also coordinated integration of testing intervals, risk stratification models, and machine learning support tools for individualized CIC monitoring.

### 5.3 Alignment With Clinical Guidelines

The alignment of candidate biomarkers with clinical guidelines reveals clear differences between established and emerging markers. Both the ESC 2022 cardio-oncology guidelines and the IC-OS consensus definitions endorse high-sensitivity troponins and natriuretic peptides (BNP/NT-proBNP) as the only biomarkers recommended for routine baseline and longitudinal monitoring, particularly in patients receiving anthracyclines, HER2-targeted agents, or immune checkpoint inhibitors [66, 68]. This supports our review’s emphasis on troponins as early indicators of subclinical myocardial injury and BNP/NT-proBNP as markers of hemodynamic stress and remodeling.

In contrast, several biomarkers discussed in this review, including sST2, galectin-3, GDF-15, and miRNAs, are not yet included in formal ESC or IC-OS recommendations. However, while these biomarkers showed promising results in early studies, further prospective validation is required before they can be incorporated into clinical guidelines. Therefore, while established biomarkers have gained clinical acceptance, emerging biomarkers remain research-oriented and represent the primary focus of future studies.

### 5.4 A Roadmap for Clinical Integration of Predictive Models

The integration of multi-parametric data into machine learning based predictive models offers a promising pathway to transition from reactive monitoring to proactive risk prediction in CIC. A proposed clinical workflow begins with the input of comprehensive patient-specific data into the machine learning model prior to or during early treatment. This data encompasses the patients’ baseline clinical profile, including age, cardiovascular history, comorbidities, and cancer type and stage; the specific treatment regimen, such as chemotherapy type and cumulative dose; serial measurements of biomarkers such as high-sensitivity troponin and NT-proBNP, alongside emerging markers; and key imaging parameters, notably echocardiographic GLS.

**Fig. 4.**
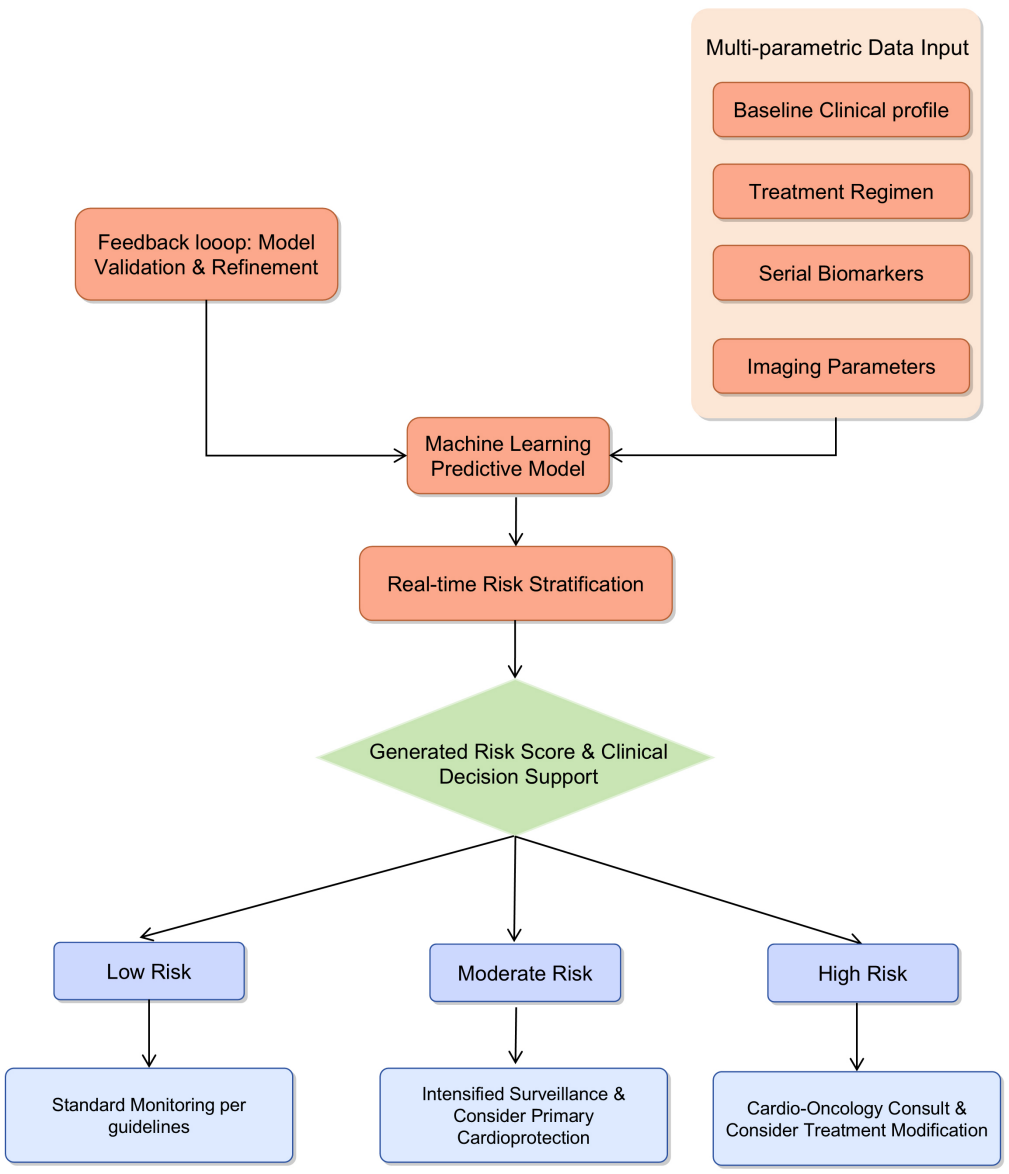
**Proposed clinical workflow for a machine learning-based predictive model in CIC monitoring**. The model integrates multi-parametric data to stratify patients into risk categories, guiding personalized monitoring and intervention strategies.

Subsequently, the machine learning algorithm synthesizes these diverse data points to generate a dynamic risk score, stratifying patients into categories such as low, intermediate, or high risk for developing cardiotoxicity. This risk assessment is then delivered to the physician within the electronic health record as a clinical decision support tool, coupled with evidence-based management suggestions tailored to each risk level. For instance, low-risk patients may continue standard monitoring, whereas intermediate-risk patients would undergo intensified surveillance and potentially receive primary cardioprotective pharmacotherapy. High-risk patients would be referred for immediate cardio-oncology consultation, with considerations for modification of chemotherapy and aggressive cardioprotection. A continuous feedback loop, in which the model is refined and validated with incoming patient outcome data, ensures the system’s ongoing optimization and accuracy. This structured approach demystifies artificial intelligence for clinicians, providing a tangible tool for personalized patient management and effectively bridging the gap between computational innovation and bedside application. The proposed clinical workflow for a machine learning-based predictive model in CIC monitoring is shown in Fig. 4.

## 6. Limitations and Future Directions

### 6.1 The Pathway to Standardization in the Use of Biomarkers

Despite encouraging progress, clinical adoption of CIC biomarkers is hindered by a lack of standardization in measurement protocols, interpretation, and clinical thresholds [7, 16]. Currently, most cardiac biomarkers such as troponins, natriuretic peptides, and fibrosis markers are evaluated using heterogeneous protocols. Variability across studies—including sampling timepoints, detection assays, and cut-off thresholds—limits comparability and weakens clinical utility [4, 36]. Uniform guidelines incorporating treatment-specific factors and patient characteristics are urgently needed. For example, changes in troponin may carry different implications depending on whether patients are receiving anthracyclines or immune checkpoint inhibitors. Only through standardized protocols can biomarkers transition from research to routine practice.

To address this critical gap, we propose a structured framework for standardization spanning the entire biomarker lifecycle. In the pre-analytical phase, consensus is needed on timing of blood sampling and standardized processing methods for both established and emerging biomarkers. The analytical phase requires the adoption of uniform, high-sensitivity assays with predefined, clinically relevant thresholds stratified by patient and treatment characteristics. Finally, the post-analytical phase should develop integrated reporting guidelines that combine biomarker levels with imaging data and clinical risk scores to provide composite risk assessments. A collaborative effort led by professional societies is essential to establish and validate this framework, thereby transforming current limitations into actionable, uniform guidelines.

### 6.2 Integration With Bioinformatics and Machine Learning

Given the complexity and heterogeneity of CIC, single markers may be insufficient for accurate prediction of risk. Machine learning and bioinformatics offer tools to integrate multi-dimensional data, including biomarkers, imaging results, genomic profiles, and clinical features, into predictive models [43]. These approaches can identify latent interactions and improve diagnostic performance beyond traditional metrics. Deep learning systems also allow for continuous updating as new data become available, enhancing long-term applicability [69].

However, barriers remain, including the need for high-quality input data, data interpretability, and clinical usability. Future work should prioritize the development of interpretable, validated AI models incorporated into electronic health records for real-time decision support.

### 6.3 Overcoming Validation Hurdles Through Multi-Center Consortia and Shared Databases

Another major limitation is the lack of large, publicly accessible, multi-center datasets dedicated to CIC research. Most current studies involve small, homogeneous cohorts from single institutions, reducing statistical power and generalizability [63]. Moreover, inconsistent outcome definitions and disparate data formats limit comparative analysis.

Establishing collaborative platforms to collect standardized biomarker, imaging, and clinical outcome data across diverse populations and treatment protocols is essential. Such databases would enable more robust validation of emerging biomarkers and support the development of comprehensive predictive models powered by machine learning [69, 70, 71, 72, 73].

The future of CIC biomarker research lies in coordinated standardization, multi-modal integration, and international collaboration, moving toward a systems-level framework that aligns molecular diagnostics with clinical decision-making. The clinical translation of exploratory biomarkers is critically limited by significant heterogeneity in studies and a pervasive lack of large-scale validation. Prevailing studies are predominantly single-center, statistically underpowered, and utilize homogeneous cohorts, which collectively limit the generalizability of their findings. To overcome these obstacles, the establishment of international research consortia and collaborative databases is paramount. We propose the creation of a dedicated CIC Biomarker Consortium, designed to prospectively collect standardized data—including clinical profiles, imaging parameters, and biobanked samples—from diverse, multi-ethnic populations across varied healthcare systems. Such an initiative must implement common data elements and uniform outcome definitions to ensure interoperability and enable pooled analyses. Furthermore, the creation of open-access biorepositories and data warehouses will facilitate independent validation of novel biomarkers and machine learning algorithms. This collaborative infrastructure will provide the necessary statistical power and population diversity to robustly evaluate biomarker performance, establish universal cut-off values, and ultimately accelerate their integration into routine cardio-oncology practice.

## 7. Future Perspectives and Conclusion

While biomarkers are indispensable for addressing the challenges associated with CIC, their full potential remains largely untapped due to persistent issues related to standardisation, validation, and integration. Advancing the field necessitates a shift towards a multiparametric approach, with emphasis on several strategic priorities. Central to this effort are the adoption of standardised operational frameworks, the facilitation of large-scale validation through global consortia, and the clinical implementation of predictive models driven by machine learning. Focused progress on these fronts will be pivotal for realizing a future in which CIC management is pre-emptive, personalised, and precise.

CIC poses a growing challenge in oncology care, particularly as cancer survival rates improve. This review highlights the current and emerging roles of cardiac biomarkers in facilitating early detection, risk stratification, and personalized monitoring strategies for CIC.

The landscape of biomarker research in CIC is broad but fragmented. Established markers such as troponins and natriuretic peptides remain useful but are limited by inconsistent thresholds and narrow diagnostic windows. Novel candidates such as inflammatory proteins, markers of fibrosis, and mitochondrial stress indicators offer deeper mechanistic insight but lack standardization and large-scale validation.

Rather than relying on isolated markers, integrative frameworks that combine molecular, functional, and clinical data are essential. Future directions include standardized monitoring protocols, large-scale multicenter validation, and incorporation of machine learning to enable individualized, real-time risk prediction. A multiparametric approach will be key to advancing precision medicine in the management of CIC.
